# Reporting of Financial and Non-financial Conflicts of Interest in Systematic Reviews on Health Policy and Systems Research: A Cross Sectional Survey

**DOI:** 10.15171/ijhpm.2017.146

**Published:** 2018-02-12

**Authors:** Lama Bou-Karroum, Maram B. Hakoum, Mira Z. Hammoud, Assem M. Khamis, Mounir Al-Gibbawi, Sanaa Badour, Divina Justina Hasbani, Luciane Cruz Lopes, Hebah M. El-Rayess, Fadi El-Jardali, Gordon Guyatt, Elie A. Akl

**Affiliations:** ^1^Center for Systematic Reviews for Health Policy and Systems Research, American University of Beirut, Beirut, Lebanon.; ^2^Clinical Research Institute, American University of Beirut Medical Center, Beirut, Lebanon.; ^3^Department of Psychiatry, Massachusetts General Hospital, Boston, MA, USA.; ^4^Faculty of Health Sciences, American University of Beirut, Beirut, Lebanon.; ^5^Faculty of Medicine, American University of Beirut, Beirut, Lebanon.; ^6^Pharmaceutical Science Master Course, University of Sorocaba, São Paulo, Brazil.; ^7^Department of Clinical Epidemiology and Biostatistics, McMaster University, Hamilton, ON, Canada.; ^8^Department of Internal Medicine, American University of Beirut, Beirut, Lebanon.

**Keywords:** Conflict of Interest, Systematic Review, Health Policy, Health Systems

## Abstract

**Background:** Systematic reviews are increasingly used to inform health policy-making. The conflicts of interest (COI) of the authors of systematic reviews may bias their results and influence their conclusions. This may in turn lead to misguided public policies and systems level decisions. In order to mitigate the adverse impact of COI, scientific journals require authors to disclose their COIs. The objective of this study was to assess the frequency and different types of COI that authors of systematic reviews on health policy and systems research (HSPR) report.

**Methods:** We conducted a cross sectional survey. We searched the Health Systems Evidence (HSE) database of McMaster Health Forum for systematic reviews published in 2015. We extracted information regarding the characteristics of the systematic reviews and the associated COI disclosures. We conducted descriptive analyses.

**Results:** Eighty percent of systematic reviews included authors’ COI disclosures. Of the 160 systematic reviews that included COI disclosures, 15% had at least one author reporting at least one type of COI. The two most frequently reported types of COI were individual financial COI and individual scholarly COI (11% and 4% respectively). Institutional COIs were less commonly reported than individual COIs (3% and 15% respectively) and non-financial COIs were less commonly reported than financial COIs (6% and 14% respectively). Only one systematic review reported the COI disclosure by editors, and none reported disclosure by peer reviewers. All COI disclosures were in the form of a narrative statement in the main document and none in an online document.

**Conclusion:** A fifth of systematic reviews in HPSR do not include a COI disclosure statement, highlighting the need for journals to strengthen and/or better implement their COI disclosure policies. While only 15% of identified disclosure statements report any COI, it is not clear whether this indicates a low frequency of COI versus an underreporting of COI, or both.

## Background

Health policy-makers are increasingly relying on systematic reviews to inform their decisions. Such reviews can provide policy-makers with robust evidence to clarify problems, frame options to address problems, and inform policy formulation and implementation.^1-3^ Policy-makers and advocacy groups have reported the use of systematic reviews in a variety of policy areas including tobacco control, traffic safety, alcohol control and perinatal care.^4,5^

Conflict of interest (COI) is defined as “a financial or intellectual relationship that may impact an individual’s ability to approach a scientific question with an open mind.”^6,7^ COIs can influence the conduct and reporting of systematic reviews resulting in misguided public policies and systems-level decisions. For instance, a study evaluating 106 review articles found that affiliation of the review author with the tobacco industry to be the only factor associated with a review concluding that passive smoking is not harmful.^8^ Another study found that systematic reviews with financial COIs were five times more likely than reviews without financial COIs to conclude there is no positive association between sugar-sweetened beverage consumption and obesity.^9^ COI may influence attitudes toward reviews themselves: a systematic survey found that opinion articles critical of the use of systematic reviews for policy-making are more likely to have industry ties than supportive articles.^10^

More recently, there has been increasing attention to non-financial COIs such as personal, political, academic, ideological, or religious COIs.^11,12^ However, it remains controversial whether non-financial COIs can affect professional judgment, and whether they should be declared and managed.^13,14^ There is one small published study that provides some evidence for the potential impact of intellectual COI on judgment. That study found that authors of primary studies with significant results are more likely than methodologists to believe that a strong association exists.^15^

Disclosure is an important first step towards identifying, assessing and responding to COIs.^6^ We are not aware of any previous studies have assessed authors’ COI disclosures in systematic reviews of health policy and systems research (HPSR). Examining the extent and the type of reporting would inform the development of guidelines addressing COI specific to the health policy and systems field. The objective of this study was to assess the frequency and types of COI that authors of systematic reviews of HPSR report.

## Methods

### Design Overview and Definitions

**Figure 1:**
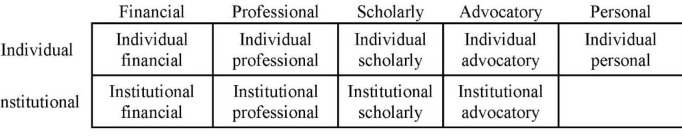
Conflicts of Interest Framework.

We conducted a cross-sectional survey. We defined a COI disclosure as a statement reporting whether a COI exists or not, under the conflict of interest section of the review articles. We classified the types of COI as per the COI framework shown in Figure 1 and detailed in Supplementary file 1. In addition to the types of COI included in the framework, we have used the word “loogly” to label “*any additional statement in the COI disclosure that attempts to downplay a disclosed relationship by suggesting that it is unrelated to COI, for example, ‘this relationship did not influence his prescription of the drug.’*”^16^ This classification was informed by a review of the literature, the International Committee of Medical Journal Editors (ICMJE) COI disclosure form and the findings from recent studies that assessed COIs reported by authors of clinical systematic reviews and randomized controlled trials.^16,17^

### Eligibility Criteria

We included systematic reviews on health policy or health systems topics published in English in 2015. We excluded policy briefs, overviews of systematic reviews, economic evaluation and costing studies, primary studies, technical reports, conference reports, proceedings, abstracts, editorials and opinion pieces.

### Search Strategy

We searched the Health Systems Evidence (HSE) database of McMaster Health Forum for systematic reviews published in 2015. HSE is a comprehensive and continuously updated repository of overviews of systematic reviews, systematic reviews, systematic review protocols, evidence briefs, economic evaluations and costing studies, health reform descriptions and health system descriptions about governance, financial, and delivery arrangements within health systems, and about implementation strategies that can support change in health systems.^18,19^ HSE relies on a number of sources to identify systematic reviews which include Medline (OVID), the Cochrane Database of Systematic Reviews, Rx for Change, Database of Abstracts of Reviews of Effects (DARE), Cochrane Qualitative and Implementation Methods Group’s reference database and Campbell Collaboration.

HSE process includes duplicate assessment of eligibility and categorization of the identified systematic reviews to the appropriate health systems arrangements (governance, financial, delivery arrangements and implementation strategies). Governance Arrangements cover topics related to Policy authority, Organizational authority, Commercial authority, Professional authority, and Consumer & stakeholder involvement. Financial arrangements include topics on Financing systems, Funding organizations, Remuneration providers, Purchasing products & services and Incentivizing consumers. Delivery Arrangements comprised topics on: How care is designed to meet consumers’ needs, By whom care is provided, Where care is provided, With what supports is care provided. Implementation Strategies cover three main topics: Consumer-targeted strategy, Provider-targeted strategy and Organization-targeted strategy.

We applied the HSE search filters to select the following categories: “systematic reviews of effects” and “systematic reviews addressing other questions.” Supplementary file 2 presents the detailed search strategy.

### Study Selection and Data Extraction

Using an online sequence generator (http://www.random.org/sequences), we drew a random sample of 200 citations from the citations captured by the search. We collected and managed study data using Research Electronic Data Capture (REDCap) tool hosted at the American University of Beirut. REDCap is a secure, web-based application designed to support data capture for research studies.^20^

We developed and pilot-tested a standardized data extraction form with detailed instructions. Data extractors completed calibration exercises and extracted data in duplicate and independently. They compared results and resolved disagreements through discussion, or with the help of a third reviewer if needed.

We extracted information on the general characteristics of the paper:

- Number of systematic review authors;
- Affiliations of the first and last authors (private or public academic institution, government, not-for-profit organization, private-for-profit, intergovernmental);
- Country of affiliation of the first author and its classification according to the World Bank list of economies issued in July 2015;
- Health policy and systems arrangements (governance, financial, delivery arrangements, and implementation strategies).

We extracted information on the characteristics of the COI disclosures:

- Form of COI disclosures (a narrative statement, an online document);
- Whether COI disclosures are made available upon request;
- Number of authors per paper who report at least one type of COI;
- Number of authors per paper who report each type and subtype of COI (see Figure 1 and Supplementary file 1);
- Characteristics of the COI (source, monetary value, duration);
- Number of authors with the same or discrepant disclosures between the main documents and the disclosures in the forms (whether published online or made available by authors upon our request);
- Whether individuals other than the authors provided COI disclosures (editors, peer reviewers, external writers, others).

We extracted information about the following characteristics of the journal:

- Impact factor
- Existence of a COI disclosure policy.

### Data Analysis

Our descriptive analyses addressed the reviews’ general characteristics, and the characteristics of the COI disclosures and provided summary data for categorical variables as frequencies and percentages. For continuous variables, application of the Kolmogorov-Smirnov (K–S) test demonstrated that the distribution of the majority of the types of COI variables was not normal. We therefore present summary data for each type of COI in a tabular format as follows:

- The percentage of systematic reviews with at least one author disclosing the specific type of COI; the denominator excludes reviews that did not include a COI disclosure statement;
- For each review, we calculated the percentage of systematic review authors reporting a specific type of COI; the denominator excludes reviews with no author reporting at least one type of COI. Then, we calculated the median and interquartile range (IQR) of these percentages across reviews.

## Results

Of the 571 systematic reviews identified by the search strategy, the 200 systematic reviews identified through random sampling were published in 152 journals.

### General Characteristics of the Included Systematic Reviews

Table 1 presents the general characteristics of the included systematic reviews. The majority addressed the topic of delivery arrangements (91%) and was conducted by authors affiliated with institutions located in high-income countries (93%). Most of the first authors (77%) and last authors (75%) were affiliated with public academic institutions.

**Table 1:** General Characteristics of the Included Systematic Reviews (N = 200)

	Overall
Number of systematic reviewers, median (IQR)	5 (3-7)
Classification of the country of the institution to which the first author is affiliated, No. (%)	
High income	185 (93)
Upper middle income	8 (4)
Lower middle income	5 (3)
Low income	2 (1)
Affiliation of first author^a^	
Public academic institution	153 (77)
Private academic institution	25 (13)
Government	22 (11)
Not-for-profit organization	13 (7)
Private-for-profit	5 (3)
Intergovernmental	0 (0)
Affiliation of last author^a^	
Public academic institution	150 (75)
Government	26 (13)
Private academic institution	23 (12)
Not-for-profit organization	17 (9)
Private-for-profit	3 (2)
Intergovernmental	1 (1)
Type of Health Systems Arrangement^a^	
Delivery arrangement	181 (91)
Implementation strategies	119 (60)
Governance arrangement	37 (19)
Financial arrangement	17 (9)

Abbreviation: IQR, interquartile range. ^a^ Systematic reviews may have more than one option that applies.

### Characteristics of the Journals

The median impact factor of the 152 journals that published the included systematic reviews was 1.92 (IQR = 1.24-3.10). Ninety-five percent (144/152) of the journals had a COI disclosure policy.

### Characteristics of the Reported Conflicts of Interest Disclosures

Eighty percent of systematic reviews (160/200) included COI disclosure statements of authors, all of which were provided narratively in the main document and none in an online form or “upon request.” Only one of the 160 reviews was published in a journal that did not have a COI policy. Of the 40 reviews that did not include a COI statement, 33 were published in journals that did have a COI policy.

Of the 160 systematic reviews that provided COI disclosure statements, 24 (15%) had at least one author reporting at least one type of COI. The two most frequently reported types of COI were individual financial COI and individual scholarly COI, 18 (11%) and 6 (4%) respectively. The median percentages of authors reporting individual financial and individual scholarly COIs were 33% and 40% respectively (out of all reviews with at least one author reporting that type of COI). The individual scholarly COIs in five reviews were related to ‘authorship of primary studies’ and in one review to ‘involvement other than authorship in primary studies’ (specifically “collaborating with one of the trial authors”).

Of the 160 systematic reviews that provided COI disclosure statements, more systematic reviews had at least one author reporting financial COIs (individual and/or institutional) compared to non-financial COIs (individual and/or institutional) (n = 18; 11% versus n = 11; 7%). Also, more systematic reviews had at least one author reporting individual COIs compared to institutional COIs (n = 24; 15% versus n = 5; 3%).

Table 2 presents the reporting by systematic review authors of the different types of COI. One systematic review reported COI by individuals other than the authors of systematic reviews, the editor.

**Table 2:** Reporting by Systematic Reviews Authors of the Different Types of Conflicts of Interest (N = 160)

	**Systematic Reviews With at Least 1 Author Reporting a Specific Type of COI**^f^ **, No. (%)**	**Distribution of the Percentage of Authors Per Systematic Review Reporting That Type of COI**^g^ **, Median (IQR)**
At least one type	24 (15)	40 (20–50)
Individual financial	18 (11)	33 (20–54)
Individual professional	1 (1)	^a^
Individual scholarly	6 (4)	40 (16–45)
Individual advocatory	0 (0)	N/A
Individual personal	0 (0)	N/A
Institutional financial	4 (3)	15 (10–42)
Institutional professional	0 (0)	N/A
Institutional scholarly	1 (1)	^b^
Institutional advocatory	2 (1)	^c^
“Other types”^h^	3 (2)	^d^
Provided a “loogly statement”	1 (1)	^e^

Abbreviations:N/A, not applicable; IQR, interquartile range. ^a^ Authors of only 1 systematic review reported individual professional COI, with the percentage being 33%. ^b^ Authors of only 1 systematic review reported institutional scholarly COI, with the percentage being 17%. ^c^ Authors of only 2 systematic reviews reported institutional advocatory COI, with the percentages being 14% and 17%. ^d^ Authors of only 3 systematic reviews reported “other types” of COI, with the percentages being 14%, 17%, and 20%. ^e^ Authors of only 1 systematic review provided a “loogly statement,” with the percentage being 43%. ^f^ One systematic review can have authors reporting more than one type of COI. ^g^ Calculated using the number of papers with at least one author reporting the specific type of COI (ie, papers counted in the preceding column) as the denominator. ^h^ “Other types” of COIs included: editorial board membership (n = 2) and ‘relationship with government agencies’ (n = 1). We consider these as individual and non-financial types of COI.

#### Individual Financial Conflicts of Interest

Table 3 presents the subtypes of individual financial COI in the 18 systematic reviews with at least one author reporting individual financial COI. The three most frequently reported subtypes were “personal fees” (n = 11; 61%), grant from source different from funding source (n =  6; 33%), and grant from source same as funding source (n = 5; 28%). The median percentages of systematic review authors reporting these three subtypes were 20%, 33%, 45% respectively.

**Table 3:** Reporting of Systematic Reviews Authors of Different Subtypes of Individual Financial (n = 18)

** **	**Systematic Reviews With at Least 1 Author Reporting the subtype of Individual Financial COI**^e^ **; No. (%)**	**Distributions of the Percentage of Authors Per Systematic Review Reporting That Subtype of COI**^f^ **, Median (IQR)**
Grant from source(s) same as funding source(s)	5 (28)	45 (23–63)
Grant from source(s) different from funding source(s)	6 (33)	33 (25–50)
Employment	4 (22)	24 (10–32)
Personal fees (other than Employment)	11 (61)	20 (17–33)
Non-monetary support	1 (6)	^a^
Drug/equipment supplies	1 (6)	^b^
Patent(s)	0 (0)	N/A
Stocks, bonds, stock options, other securities	2 (11)	^c^
“Other subtypes”	2 (11)	^d^

Abbreviations:N/A, not applicable; IQR, interquartile range. ^a^ Authors of only 1 systematic review reported “non-monetary support” COI, with the percentage being 14%. ^b^ Authors of only 1 systematic review provided a “Drug/equipment supplies,” with the percentage being 17%. ^c^ Authors of only 2 systematic review reported “Stocks, bonds, stock options, other securities” COI, with the percentage being 14% and 50%. ^d^ Authors of only 2 systematic review reported “Other subtypes” COI, with the percentage being 25% and 20%. ^e^ One systematic review can have authors reporting more than one type of COI. ^f^ Calculated using the number of papers with at least one author reporting the specific type of COI (ie, papers counted in the preceding column) as the denominator.

Supplementary file 3 presents the characteristics of the reported individual financial COI of systematic reviews. Of the 18 systematic reviews with at least one author reporting individual financial COI, 16 reported the source of financial COI, only one of which specified the relationship of the source to the field under study. In that case, the source produced a product not the subject of the review but in the same field. Three reviews reported on the timing of the conflict as during the conduct of the study. None of the reviews reported on the monetary value of the financial COI.

## Discussion

### Summary of Findings

In summary, four of five HPSR systematic reviews included a COI disclosure statement. Only 15% of systematic reviews including a disclosure had at least one author reporting at least one type of COI. The two most frequently reported types of COI were individual financial COI and individual scholarly COI (Table 2). Institutional COIs were less commonly reported than individual COIs while non-financial COIs were less commonly reported than financial COIs (Table 2).

### Strengths and Limitations

This study used a comprehensive COI framework including financial, non-financial and institutional COIs. We developed the framework based on a review of the literature, the ICMJE COI disclosure form and the findings from recent studies that assessed COIs reported by authors of clinical systematic reviews and clinical trials.^16,17^ One potential limitation is that we drew our sample from only one database, the HSE. However, HSE is a comprehensive and continuously updated database that draws its content from major sources of systematic reviews.^18^

### Comparison to Similar Studies

Our findings suggest that COI disclosures statements are less frequently included in systematic reviews of HPSR compared to systematic reviews in other fields. Hakoum et al found 97% of clinical systematic reviews and 94% of clinical trials to include COI disclosure statements.^16,17^ This less stringent requirements for COI disclosure in journals publishing HPSR systematic reviews versus those publishing clinical systematic reviews is reflected in the field in general: while 99% of Core Clinical Journals have a COI disclosure policy,^21^ 93% of HPSR journals have such a policy.^22^

All COI disclosures were in the form of a narrative statement in the main document and none in an online document. This reflects the absence of a standard – such as the ICMJE form for medical journals – COI disclosure form for journals publishing HPSR. Indeed, our team found that only 7% of HPSR journals were members of the ICMJE and 88% of journals require COI disclosure in a narrative statement.^22^

**Figure 2:**
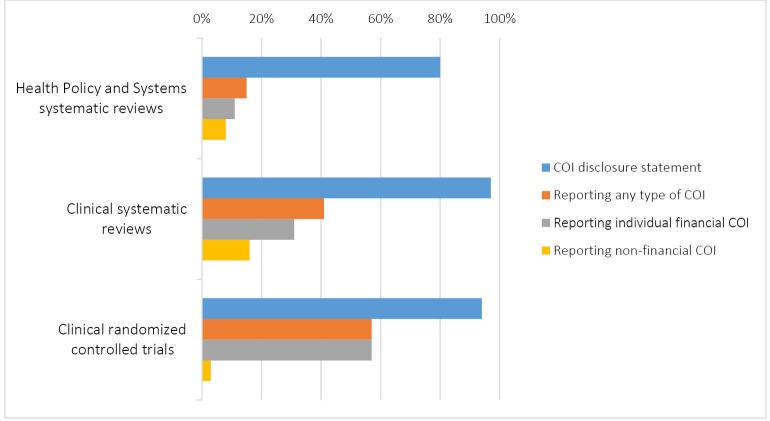
Health Policy and Systems Research Systematic Reviews’ Reporting of Conflicts of Interest Characteristics Compared With Those of Clinical Systematic Reviews and Randomized Controlled Trials.

The included HPSR systematic reviews had a far lower frequency (15%) of authors reporting any type of COI than clinical systematic reviews (41%) or clinical trials (57%) (See Figure 2).^16,17^ This may be a consequence of the HSPR journals’ lack a standard COI disclosure form making it less likely that authors of HPSR recognize their COIs. Another possible interpretation is that HPSR systematic reviews authors may simply have less COIs, mainly financial, than authors of trials.

Generally, more systematic reviews had at least one author reporting financial COIs compared to non-financial COI. Also, more systematic reviews had at least one author reporting individual COIs compared to institutional COI. This finding is consistent with findings in clinical systematic reviews and clinical trials.^16,17^ However, across all reviews, the reporting of non-financial COI in HPSR systematic reviews (7%) is higher than in the Core Clinical journals systematic reviews (2%).^16^ One potential explanation is the higher percentage of HPSR journals (81%) asking authors to disclose their non-financial COIs compared to Core Clinical journals (57%) and Oncology journals (42%).^21-23^ Apparently, journals in the HPSR field are more aware of the importance of non-financial COIs.

Only one systematic review reported the COI disclosure by individuals other than the authors of systematic reviews, namely the editor. No systematic review reported the COI disclosure by the reviewers. It is possible that editors and reviewers disclosures are collected but not published.

The majority of included systematic reviews were conducted by authors affiliated with institutions in high income countries, a finding consistent with the small proportion of systematic reviews from low and middle income countries in general.^24-27^ We also found that the majority of systematic reviews address the delivery arrangements topic, which corroborates the findings of previous studies on the topics covered by HPSR systematic reviews.^24-26^

### Implications for Research, Policy and Practice

Given that 20% of systematic reviews did not have COI disclosure statements, HPSR journals should consider strengthening and/or better implementing their COI disclosure policies. The fact that the majority of those reviews were published in journals with existing COI policy, highlights the need for journals to implement their policies. As a small percentage of authors report specific types of COI in this study, it would be important for future studies to verify the completeness and accuracy of authors’ disclosures and to investigate the existence of under-reporting in this field. It is also important to keep in mind that a COI disclosure does not necessarily imply bias, and there is a need to develop valid methods to better judge when a disclosed COI is likely to be associated with bias.

HPSR journals should recognize the importance of COI disclosure by reviewers and editors and improve their collection and reporting. Publicizing the COI disclosure of reviewers and editors can increase the credibility of the journal and the trust of the readers and mitigate any potential associated bias.

The disclosure and management of COIs helps increase the credibility and trust in research not only by the public but also by policy-makers. Disclosure represents a first step in transparency that allows the consideration that conflicts may have influenced conduct of a review and the conclusions that review authors present

## Ethical issues

The study involves no human subjects and required no ethical approval.

## Competing interests

Authors declare that they have no competing interests.

## Authors’ contributions

LBK, MBH, FEJ, GG, and EAA conceived and designed the study. LBK coordinated the study throughout. EAA had full access to all of the data in the study and takes responsibility for the integrity of the data and the accuracy of the data analysis. LBK and MBH ran the search and study selection processes. LBK, MZH, AMK, MAG, SB, DJH, LCL, and HER extracted the data. LBK, AMK, and EAA analysed and interpreted the data. LBK and EAA wrote the first draft of the manuscript. All authors critically revised the manuscript and approved the final version.

## Funding

This project was funded by the American University of Beirut Faculty of Medicine’s Medical Practice Plan (MPP) funds.

## Authors’ affiliations

^1^Center for Systematic Reviews for Health Policy and Systems Research, American University of Beirut, Beirut, Lebanon. ^2^Clinical Research Institute, American University of Beirut Medical Center, Beirut, Lebanon. ^3^Department of Psychiatry, Massachusetts General Hospital, Boston, MA, USA. ^4^Faculty of Health Sciences, American University of Beirut, Beirut, Lebanon. ^5^Faculty of Medicine, American University of Beirut, Beirut, Lebanon. ^6^Pharmaceutical Science Master Course, University of Sorocaba, São Paulo, Brazil. ^7^Department of Clinical Epidemiology and Biostatistics, McMaster University, Hamilton, ON, Canada. ^8^Department of Internal Medicine, American University of Beirut, Beirut, Lebanon.

## Supplementary files

Supplementary file 1. The types of COI.

Supplementary file 2. Search strategy.

Supplementary file 3. The characteristics of the reported individual financial COI of systematic reviews.

## Key messages

**Implications for policy makers**

- Health policy and systems research (HSPR) journals should strengthen and/or better implement their conflicts of interest (COI) disclosure policies.
- The disclosure and management of COI may help increase the credibility and trust in systematic reviews by policy-makers.
- Given the potential influence of COI on research, practice and policy, governments and funding agencies are called to support research in this field.

**Implications for the public**

Disclosure represents a first step in transparency that allows readers to consider whether conflicts may have influenced conduct of a review and the conclusions that review authors present.
