# Supplementary material for: Reporting of Financial and Non-financial Conflicts of Interest in Systematic Reviews on Health Policy and Systems Research: A Cross Sectional Survey
Source: Int J Health Policy Manag. 2018 Feb 12;7(8):711–7. doi: 10.15171/ijhpm.2017.146 (PMC6077276; doi:10.15171/ijhpm.2017.146)
Supplement: Supplementary file 1 — The types of COI. [file ijhpm-7-711-s001.pdf]

Supplementary 1: Conflicts of Interest

Part 1: Classification of Conflicts of Interest

	Financial	Professional	Scholarly	Advocatory	Personal
Individual	Individual financial	Individual professional	Individual scholarly	Individual advocatory	Individual personal
Institutional	Institutional financial	Institutional professional	Institutional scholarly	Institutional advocatory	
	Direct benefit	Benefit through professional status			
		Clinical	Non-clinical		
			No personal opinion stated	Personal opinion stated	
				Non-private	Private

COIs can be individual or institutional.

- Individual COIs arise from relationships established by the individual. Types of individual COIs include financial, professional, scholarly, advocatory and personal.
- Institutional COIs arise from relationships established by the individual's institution, and of which that individual is aware. Types of institutional COIs include financial, professional, scholarly and advocatory.

Part 2: Classification of Individual Financial COI

Definition:<br>Individual Financial COI arises from any payment, research funding, consultancy, advisory board membership, and the like from the manufacturer of a drug or device under consideration. This type of COI may involve the individuals themselves, their families or a business they own. Typically, a timeframe of three to five years is considered for these COIs.

Grant from source(s) same as funding source(s)	*There could be a differentiation whether the grant is going to the investigator or to his/her institution.*<br>*Types: grant; salary for research; contract; fellowship; unrestricted educational funding; peer-reviewed grant funding*<br>*e.g. a paper is funded by AHRQ and an author received a grant from AHRQ, during conduct of the study or when unrelated to the submitted work or when not specified*
Grant from source(s) different from funding source(s)	*e.g. a paper is not funded, or a paper is funded by AHRQ, but an author received a grant from Pfizer*
Employment	*Types: former employment; current employment; stipend; salary*
Personal fees (other than Employment)	*Types: honoraria, royalties, fees for consulting, lectures, speakers' bureaus, expert testimony, presentations, editorial work, manuscript preparation, trial involvement. management, educational support, production of books, article research, scientific meetings, entertainment, gift, charitable contribution, other affiliations (e.g. advisory board, steering committee membership)*
Non-monetary support	*Types: travel paid; writing assistance; administrative support; food and beverage*
Drug/equipment supplies	
Patent(s)	
Stocks, bonds, stock options, other securities (e.g. equity)	
Other forms	

Part 3: Classification of Individual Professional COI

Definition:<br>Individual Professional COI arises when an individual (1) receives financial benefit through professional status, (2) is "engaged in a specified activity as one's main paid occupation", or (3) "is a member of a professional group of individuals."
e.g. an author that is a dietician conducts a study looking at advice vs. no advice from a dietician; an author that provides colonoscopy services; an author that works at a warfarin clinic

Part 4: Classification of Individual Scholarly COI

Definition:<br>Individual Scholarly COI arises when an individual participates in scholarly activities related to the issue under consideration. By conducting scholarly work on a specific topic, there is a potential that the individual becomes intellectually committed to the findings of that work.	
Authorship of primary studies	*e.g. trials; case-control studies*
Authorship of secondary studies	*e.g. systematic reviews*
Involvement in primary studies (but not authorship)	*e.g. collaborated in a trial; provided advice on a protocol; commented on a manuscript; medical monitor of a trial; site or local investigator of a study*

Part 5: Classification of Individual Advocatory COI

Definition:<br>Individual Advocatory CO arises when an individual has taken a position or has an opinion and expresses it in a public statement.	
Expressed an opinion in an editorial	
Expressed an opinion in a public statement (that is not an editorial)	*e.g. meeting poster or presentation; grand rounds talk*
Expressed an opinion in published work	*e.g. textbook; review article*
Participating in a guideline panel	*e.g. Chair of American Heart Association Get With The Guidelines Steering Committee*

Part 6: Classification of Individual Personal COI

Definition:<br>when an individual has personal opinions or conditions that concern one's private life, relationships, and emotions rather than one's career or public life.	
Beliefs (religious, political, philosophical)	*e.g. an author against organ donation or abortion attributed to personal religious beliefs*
Personal characteristics (gender, age, race, physical/psychiatric condition, sexual orientation)	*e.g. an author with a physical disability conducting a study on the benefit of physical rehabilitation*

Part 7: Classification of Institutional Financial COI

Definition:<br>Institutional Financial COI arises when an institution to which an individual belongs has a relationship with the manufacturer of a drug or device under consideration. Such institutions include academic medical centers and professional societies.	
Seeking and receiving gifts, endowments, or grants from companies, for example, a gift of an endowed university chair	*Types: grants for research/fellowship/salary support; merit awards; endowments; patent funds; educational fees; funds for author activities (speaker fee, consultancy, honoraria, board membership, testimony, writing); funds for drug/equipment supplies*
Conduct of research within the institution that relates to the issue under consideration and could affect the value of the institution's patents or its equity positions or options in biotechnology, pharmaceutical, or medical device companies	
Senior officials who act on behalf of the institution have personal financial interests related to the issue under consideration	

Part 8: Classification of Institutional Professional COI

Definition:<br>when an institution employs professionals who advocate for clinical services related to the issue under consideration but don't provide those services themselves.

Part 9: Classification of Institutional Scholarly COI

Definition:<br>Institutional Scholarly COI arises when an institution/organization focuses on a specific research topic or funds research on a specific topic resulting in a possibility of having certain views on the topic.	
Conducts primary/secondary studies	*e.g. an author works at a hospital which is enrolling participants in a trial on a certain topic; an author is a member of an organization that has a research focus on a certain topic*
Funds primary/secondary studies	

Part 10: Classification of Institutional Advocatory COI

Definition:<br>Institutional Advocatory COI arises when an individual (paid employee or unpaid member) belongs to an institution/organization that:	
is an advocacy group that clearly advocates for the issue under consideration	*e.g. an author is an unpaid Board Member of Support and Advocacy for Autism Spectrum Individuals and Families*
has advocacy related to the issue under consideration as part of its mission, objectives, work, or stated position (i.e. position statement, editorial, blog, amicus brief, or legislature or legal testimony)	*e.g. an author is a member of the Veterans Affairs Rheumatology Field Advisory Committee*
shows "public support for or recommendation of a particular cause or policy"	
has senior officials who act on its behalf and have COI related to the issue under consideration	
