# Supplementary material for: Reporting of Financial and Non-financial Conflicts of Interest in Systematic Reviews on Health Policy and Systems Research: A Cross Sectional Survey
Source: Int J Health Policy Manag. 2018 Feb 12;7(8):711–7. doi: 10.15171/ijhpm.2017.146 (PMC6077276; doi:10.15171/ijhpm.2017.146)
Supplement: Supplementary file 2 — Search strategy. [file ijhpm-7-711-s002.pdf]

Supplementary 2: Search strategy

1. Go to https://www.healthsystemsevidence.org/
2. Go to “Advanced Search”
3. Filter documents by domain
4. Go to “system arrangements” and choose “any system arrangement”
5. Go to “implementation strategies” and choose “any implementation strategies”
6. Filter documents by data range “2015-2015”
7. Filter documents by type and choose “systematic reviews of effects” and “systematic reviews addressing other questions”
