# Supplementary material for: Reporting of Financial and Non-financial Conflicts of Interest in Systematic Reviews on Health Policy and Systems Research: A Cross Sectional Survey
Source: Int J Health Policy Manag. 2018 Feb 12;7(8):711–7. doi: 10.15171/ijhpm.2017.146 (PMC6077276; doi:10.15171/ijhpm.2017.146)
Supplement: Supplementary file 3 — The characteristics of the reported individual financial COI of systematic reviews. [file ijhpm-7-711-s003.pdf]

Supplementary 3. Characteristics of the reported individual financial conflicts of interest (COI) of systematic reviews (N=18)

	**Overall**<br><br>**No. (%)**
Disclosures that specify the following characteristics of the individual financial COI:	
Source	16 (89%)
Specify whether a source produces one of the interventions subject of the SR §	0 (0%)
Specify that a source produces interventions not subject of the SR but under the same field §	1 (6%) §
Monetary value	0 (0%)
Duration ¶	3 (17%)
During conduct of the study	3 (100%) ¶
1 year	0 (0%)
2 years	0 (0%)
3 years	0 (0%)
4 years	0 (0%)
5 years	0 (0%)
More than 5 years	0 (0%)

§ Calculated using N=16 as the denominator in the overall group. The disclosure included the following: "*Prof Singh reports a British Lung Foundation grant for RCT of self-management support post admission.*"
¶ Calculated using N=3 as the denominator in the overall.
